# Evolutionary Explanations of the “Actuarial Senescence in the Wild” and of the “State of Senility”

**DOI:** 10.1100/tsw.2006.209

**Published:** 2006-08-31

**Authors:** Giacinto Libertini

**Affiliations:** Caivano 80023, Naples, Italy

**Keywords:** actuarial senescence, age-associated diseases, age changes, aging, evolution, mortality rate, senescence

## Abstract

A large set of data suggests that progressive reduction of fitness and senile decay in vertebrates are in correlation with the decline of cell replication capacities. However, the limits in such capacities are hardly explained in evolutionarily terms by current gerontological theories that rule out fitness decline as something genetically determined and regulated, and therefore somehow favored by natural selection.Four theories are tested as possible explanations of the “increasing mortality with increasing chronological age in populations in the wild” (“IMICAW”[1]), alias “actuarial senescence in the wild”[2], and of the observed negative correlation between extrinsic mortality and the ratio between deaths due to intrinsic mortality and deaths due to extrinsic mortality. Only the theory attributing an adaptive value to IMICAW allows an evolutionary explanation for it and for the aforesaid inverse correlation, while the other three theories (“mutation accumulation”, “antagonistic pleiotropy”, and “disposable soma” th.) even predict a positive correlation.Afterwards, the same theories are tested as possible explanations for the “state of senility”[3], namely the deteriorated state of individuals in artificially protected conditions (captivity, civilization, etc.) at ages rarely or never observable in the wild. With the distinction between “damage resulting from intrinsic living processes”[4], alias “age changes”[5], and “age-associated diseases”[4,5], the same theory explaining IMICAW allows a rational interpretation of the first category of phenomena while another theory, the “mutation accumulation” hypothesis, gives an immediate interpretation for the second category.The current gerontological paradigm explaining the increasing mortality with increasing chronological age as consequence of insufficient selection should be restricted to the “age-associated diseases”. For IMICAW, it should be substituted with the concept of a physiologic phenomenon genetically determined by a balance of opposite selective pressures — strictly in terms of kin selection — and, for “age changes”, with the action of the same IMICAW-causing mechanisms at ages when selection becomes ineffective.

## INTRODUCTION

It is now well defined that Hayflick limit[6,7] is in correlation with the shortening of telomeres with aging and that an enzyme, telomerase, lengthens telomeres and balances terminal DNA losses caused by DNA polymerase.

Regulatory proteins repress telomerase[8]. Disrupting telomerase action causes telomere shortening and reduction of potential growth[9] and, on the contrary, activation of telomerase causes telomere lengthening and immortalizes the cells[10,11].

The easy simplification that cells with activated telomerase have an unlimited duplication capacity, while cells with inactivated telomerase show a limited duplication capacity strictly proportional to telomere length, is imperfectly supported by empirical data and a more sophisticated and realistic model has been suggested in a review by Blackburn[12]. Indeed, if somatic cell growth potential is strictly proportional to telomere length, it would be totally unimpaired up to a critical length, while under this length, namely starting from a certain number of duplications, there would be a sudden slump of the growth potential. But, cell populations show a progressive reduction of growth potential starting from early ages, that is, for single cells, even with telomeres having the maximum length, the passage from "cycling state" (duplication possible) to "noncycling state" (duplication impossible) is stochastic[13,14]. In the aforesaid review, it is suggested ("Blackburn's hypothesis") that telomere, a dynamic nucleoprotein complex, can switch stochastically between two states: capped and uncapped. Capping preserves telomere physical integrity, allowing cell division to proceed. Uncapping occurs normally in dividing cells, regardless of telomere length, but the probability of returning to the capped state is proportional to telomere length and the uncapped state, if left uncorrected too long, elicits the passage to the noncycling state.

To contrast the possible objection that species, such as the mouse, with long telomeres have precocious aging, it is necessary to point out that Blackburn's hypothesis does not require, for different species, a fixed ratio between telomere length and stability of telomeric nucleoprotein complex, being easily assumable that telomere function in its modulation is different for each species, as already documented[15].

However, according to this model, and with the support of the aforesaid observations, a cell population, all cells with inactivated telomerase and initially with telomeres at their maximum length, shows, from the beginning, a gradual reduction of duplication capacity, at first minimal, afterwards more and more growing. Moreover, it is interesting to note that even cells having telomerase activated and therefore always telomeres with maximum length should show a small percentage of cells that at each division pass to the noncycling state and since, in addition, stem cells, unlike germ cells, have levels of telomerase activity such to stabilize only partially telomere length[16], therefore, *in vivo* stem cells could not replace forever differentiated elements in cell populations that are in renewal[15].

Cell replication capacity is indispensable for cell turnover that is a general pattern in vertebrates: "Each day, approximately 50 to 70 billion cells perish in the average adult because of programmed cell death (PCD). Cell death in self-renewing tissues, such as the skin, gut, and bone marrow, is necessary to make room for the billions of new cells produced daily. So massive is the flux of cells through our bodies that, in a typical year, each of us will produce and, in parallel, eradicate a mass of cells equal to almost our entire body weight"[17].

For many cell types, PCD is completed via detachment from the somatic surface (e.g., skin, gut) or with the removal by macrophages (e.g., red cell). For other tissues and organs, cells are usually eliminated with the mechanism of apoptosis. Indeed, apoptosis was described for the first time and clearly differentiated from necrosis in the observation of normal liver epatocytes[18], and is an essential phenomenon for cell turnover in adult organs[19,20,21], e.g., adipocytes[22]; biliary epithelial cells[23]; bone[24]; cartilage[25]; kidneys[26]; liver[27]; lungs[28]; gliocytes[13]; prostate[19]; skeletal muscle[29,30]; thyroid[31].

In short, at least for vertebrates, three categories of cells are currently distinguished:

1. With high turnover, e.g., intestinal crypts cell[32].
2. With moderate turnover, e.g., cell of the deep layers of skin and endothelial cells[33], heart myocytes[34], muscle myocytes. (Stem cells from muscles of old rodents divide in culture less than cells from muscles of young rodents[35]; a transplanted muscle suffers ischemia and complete degeneration and then there is a complete regeneration by action of host myocyte stem cells that is poorer in older animals[36].)
3. With no turnover, e.g., neurons, with a few exceptions[37], but always with metabolic dependence on gliocytes that are cells with turnover.

A simple spontaneous hypothesis about the mechanisms underlying pathophysiological alterations in old vertebrate individuals, viz. about "damage resulting from intrinsic living processes"[4] alias "age changes"[5], has been inferred: The more or less precocious aging is the consequence of the less or greater genetically determined cell replication capacity and of the related cell senescence (Fossel's "cell senescence general model of aging"[15]).

This hypothesis is not at all new. Disregarding Weismann's intuitions ("Weismann's arguments led him to propose that the physiological decline which occurs in senile animals results from an evolved limitation to the reproductive potential of somatic cells"[38]; "[Weissmann] was likewise dubious about the exact nature of the death-mechanism, but indicated that it might involve a specific limitation on the number of divisions that somatic cells might undergo."[3]), Hayflick, from the beginning of his observations, already suggested that cell replication limits could be a model for the study of aging[7] and later confirmed his suggestion: "... if normal animal cells do indeed have only a limited capacity for division in cell culture, then manifestations of aging might very well have an intracellular basis."[39]. In effect, telomere relative shortening, a strict intracellular event, is currently thought to contribute to aging[15,33], but this approach has been always opposed by the fact that aging caused by genetically regulated mechanisms is in total contrast with current theories on senescence[40]. The same Hayflick has disavowed his old suggestion[5]! Another difficulty was that without Blackburn's hypothesis, or equivalent mechanisms, the reduction of cell replication capacities would be abrupt at a certain age and therefore "age changes" should manifest themselves in a very limited period and not, as observed, slowly, with alterations initially and for a long time by no means defined as senescence. In effect, the curve of the reduction of cell duplication capacity — well explained by Blackburn's hypothesis — mimics the life tables of species in the wild that show increment of mortality with increasing age.

However, if the hypothesis is true, tissues having cells with reduced growth potential should be characterized by: (a) reduced number of cells (atrophy), (b) reduced cell turnover, (c) possible substitution of missing specific cells with nonspecific cells, (d) hypertrophy of the remaining specific cells, (e) altered functions of cells with shortened telomeres or definitively in noncycling state (cell senescence)[15], (f) vulnerability to cancer as a consequence of dysfunctional telomere-induced instability[41].

These "alterations by reduced duplication capacity" ("ARDC") in the case of dysfunctions of stem cells in cycling state or of somatic cells in cycling state should be observable respectively in cells with high and moderate turnover rate, while in the case of the "state of senility"[3] they should be observable with all cells.

As a matter of fact, dyskeratosis congenita, an inherited human disease[42], is an excellent model of dysfunctions of stem cells in cycling state[33] whereas a prototype of dysfunctions of somatic cells in cycling state is Werner syndrome, as illustrated in a review[43]. Marciniak and Guarente[33] have skillfully expressed the crucial difference between the two syndromes.

Limiting the argument to the human species because of the huge quantity of available data, if the hypothesis is true, very old individuals, that is, those who demonstrate "age changes" in their most extreme form, excluding "age-associated diseases" and damages by extrinsic factors (categories 2 and 3, respectively, in Masoro's 1998 classification[4]), should show widespread and pronounced signs of ARDC for all organs and tissues.

The simple careful reading of a trustworthy textbook of geriatrics and gerontology gives a detailed confirmation of this prediction (e.g., for skin[44]). Some data are available from a long time ago (e.g., for small bowel[45]). A modern, very-documented review from the viewpoint of telomere-telomerase regulations has been published[15].

In support of Fossel's two main arguments:

1. The number of circulating endothelial progenitor cells, from which the effectiveness of endothelial repair is conditioned, has been shown to be inversely age related, reduced by known vascular risk factors and increased by drugs, such as statins, that are believed to protect organ integrity, and appears to predict cardiovascular risk better than Framingham risk score[46,47].
2. To oppose the correct objection that the hypothesis is insignificant for cells with no turnover:
   A. "Many investigators have emphasized post-translational alterations of long-lived crystalline proteins as the basis for senescent ocular cataracts. It is apparent in Werner syndrome that the cataracts result from alterations in the lens epithelial cells."[43] and this is coherent with age-related reduction in growth potential for lens epithelial cell reported for normal human subjects[48].
   B. Retina cones and rods, highly differentiated nervous cells with no turnover, for their survival need the macrophagic activity of retina pigmented cells, highly differentiated gliocytes, that show turnover, decline with age, and whose insufficient action causes retina macular degeneration, a disease which is commonly age related[49].

In short, cell replication capacity is genetically determined by highly sophisticated and regulated mechanisms, and is an essential and integral part of cell turnover, another highly sophisticated and regulated phenomenon. Its slow down, together with consequent cell senescence alterations, appears to be the main cause of "age changes". All this has little plausibility as a consequence of mutations, pleiotropic genes, or of insurmountable constraints, considered by current gerontological thought as the basic determinants of aging, but is in accordance with the idea of a programmed progressive reduction of fitness. Yet, this conception implicitly requires that genetically determined and regulated mechanisms causing this reduction of fitness are somehow favored by natural selection, a possibility absolutely excluded by current theories about aging[40].

The evident contradiction is not underlined by the experts in the limits of cell turnover and in cell senescence[15] and, on the other side, evolutionary biologists, attempting an explanation for senescence in terms of selection or of its failure, do not give an evolutionary justification of the above-mentioned limits.

The possible objection that telomerase restrictions have the adaptive meaning of limiting cancer risks does not explain their different modulation in the various species and the existence of "animals with negligible senescence" (see later in the relative section).

Concerning this problem, this paper tests three current theories on aging and a theory that predicts the increment of mortality in the wild as a programmed phenomenon favored by selection in certain conditions.

## TOPIC

We first need to state two definitions ("IMICAW" and "state of senility") and a non-definition ("senescence"/"aging"), since without these specifications the arguments cannot be correctly illustrated and perceived:

- **Definition 1** — "IMICAW", acronym of "increasing mortality with increasing chronological age in populations in the wild"[1], alias "actuarial senescence in the wild"[2] is a real phenomenon[2,50,51,52,53,54], and by definition, according to its presence in the wild, is subject to natural selection. IMICAW describes an actuarial phenomenon and is not at all a synonym of aging.

- **Definition 2** — The "state of senility"[3] is the deteriorated state of individuals in artificially protected conditions with low mortality (captivity, civilization, etc.) at ages rarely or never observable in the wild, namely the state of individuals with age-related reduced fitness to wild conditions smaller than an arbitrarily established value. By definition, the "state of senility", rare in the wild because of its reduced fitness, is largely an artificial state and is minimally subject to natural selection. For a non-IMICAW species, i.e., that in the wild does not show a decrement of fitness with age (disregarding mortality increment due to the effects of the accumulation of injury damages), by definition, individuals in the "state of senility" are nonexistent in the wild.

Fig. 1 illustrates the aforesaid definitions.

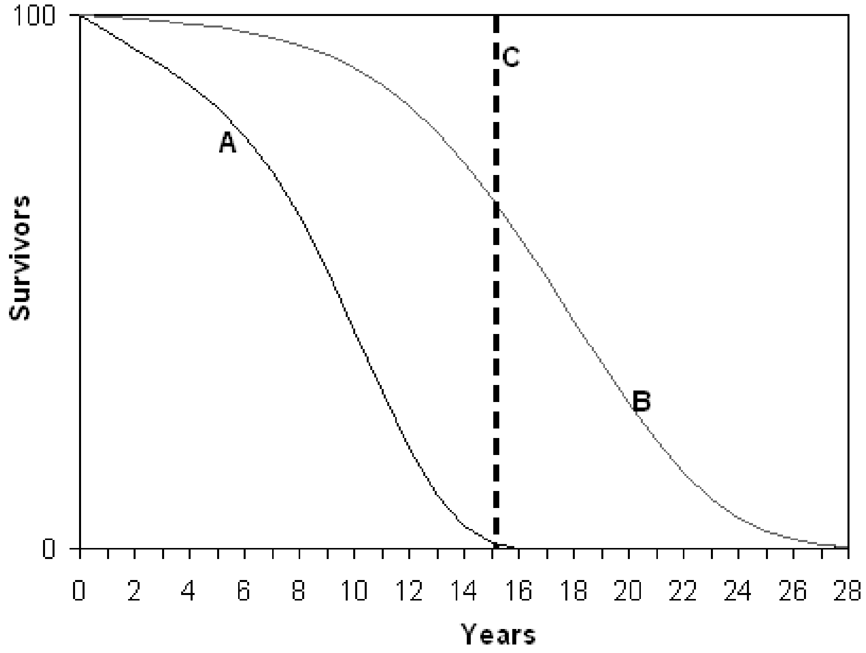

**FIGURE 1.** Curve A: Life table of an IMICAW species in the wild (Weibull's equation has been used; data, from Ricklefs[54], are of *Panthera leo*: $m_o = 0.032$, $\alpha = 2.52E-4$, $\beta = 3$); curve B: life table of the same species in artificial conditions of arbitrarily lowered mortality (= 1/8 of mortality in the wild); line C: arbitrarily defined line marking the beginning of the "state of senility" (at the time when, in the wild, reduced fitness has become smaller than an arbitrarily established value). The fraction of individuals surpassing this line is small in the wild and the grade of their functional decay is in the arbitrarily defined range of the "state of senility". With artificially lowered mortality, this fraction becomes appreciable. It has to be underlined that for non-IMICAW species, namely for species where intrinsic mortality does not increase with age, viz. where fitness does not decrease with age, individuals in the state of senility are nonexistent in the wild and the concept of line C is inapplicable.

- **A nondefinition** — "Ageing is usually defined as the progressive loss of function accompanied by decreasing fertility and increasing mortality with advancing age"[40]. "Senescence (or aging) is a decline in physiological functioning with age that results in a decrease in reproductive rate, increase in mortality rate, or both (Finch, 1990; Abrams, 1991; Rose, 1991; Holmes and Austad, 1995b)"[54]. In both these practically identical definitions, there is the noteworthy absence of the restriction "in the wild". But, while in the first paper we read: "… there is scant evidence that senescence contributes significantly to mortality in the wild … As a rule, wild animals simply do not live long enough to grow old…", in the second paper, that is cited in the first as relevant argument supporting current theories on "aging", "actuarial senescence in the wild" is investigated and we read: "Senescence is an important source of mortality in natural populations…".

In shorts, "senescence" is used in the first case as synonym of the "state of senility" and in the second as synonym of IMICAW, namely, a single term is used for two different concepts.

"In fact, 'ageing' is used with so many different meanings in so many different contexts that it is sometimes highly confusing when used without proper qualification."[38]. Therefore, to avoid possible ambiguities the term "senescence"/"aging" will be written in quotation marks to emphasize its poorly defined meaning, and will be used only in quoting from current theories on "senescence".

The hypothesis to be tested is that the paradigm of the increasing mortality with increasing chronological age only as consequence of harmful genes or other agents insufficiently countered by natural selection is criticizable both with theoretical and observational arguments. Moreover, that it is possible to partially replace it with the opposite concept that a genetically determined reduction of the mean duration of life ("ML") in the wild is evolutionarily advantageous in certain conditions.

The object of the next two paragraphs is to evaluate four different theories as possible evolutionary explanations of IMICAW and, afterwards and separately, of the "state of senility".

The first three theories are current hypotheses on the causes of "senescence"[40,54]:

- **Theory A - "mutation accumulation" theory** — "Aging" is due to the effects of harmful mutations, accumulated over evolutionary time, which manifest themselves at older ages when, in the wild, survivors are very few or absent and, consequently, selective forces are too weak to eliminate them[55,56,57,58,59].
- **Theory B - "antagonistic pleiotropy" theory** — "Senescence" is caused by pleiotropic genes with beneficial effects at early ages and deleterious effects at later ages[3,60]. This theory implicitly assumes that hypothesized genes have no alternative gene with analogous good effects at early ages and no deleterious effect at later ages.
- **Theory C - "disposable soma" theory** — "Aging" has environmental or somatic and not genetic causes, and evolutionary responses to them are increasingly limited at older ages by physiological, biochemical, or environmental constraints. So, in the evolutionary search of an optimal allocation of metabolic resources between somatic maintenance and reproduction, the first is sacrificed[61,62]. As for theory B, it is necessary to postulate that hypothesized constraints have no alternative with analogous advantages at early ages and no disadvantage at later ages.
- The fourth theory, **theory D - "adaptive IMICAW" theory**[1] — An evolutionary interpretation of IMICAW as an adaptive phenomenon selectively advantageous in certain conditions and in terms of inclusive fitness. This theory is explained in brief in the next paragraph but, since it is radically different from the other three theories and in contrast with the current gerontological thought, the reading of the original paper is advised to avoid misunderstandings.

## THE FOUR THEORIES TESTED AS EXPLANATION OF IMICAW

In this section, the four theories are tested exclusively as a possible explanation of IMICAW and by no means as a possible explanation of the "state of senility".

### Theory A

Against theory A as possible explanation of IMICAW, it has to be remarked that: "As pointed out by Mueller and Rose (1996), at some point selection becomes weaker than the forces of mutation and genetic drift and adaptive evolutionary responses cease ... However, this point occurs well after 99% of individuals in a population have died, and the mechanism therefore is not relevant to mechanisms of senescent death occurring up to this point”[54].

A possible objection is that Mueller and Rose’s statement could be dubious in the case of the combined action of many harmful genes. In regard to this possibility, a simple theoretical argument — that should not be confused with theory D! — was formulated[1].

In a non-IMICAW population, the life table of which is given by the equation:

$$Y_t = Y_0 \cdot (1 - \lambda)^t \qquad (1)$$

where $Y_0$ = starting population, $Y_t$ = survivors at time t, $\lambda$ = death rate, if C is a dominant allele expressing its disadvantageous action (S) only and exclusively at the time t (“t-gene”), C' its neutral allele, V mutation rate of C' in C (while mutation rate of C in C' is considered negligible), then the frequency of C at the (n + l)th generation is given by:

$$C_{n+1} = \frac{C_n \cdot (1 - S \cdot Y_t - V) + V}{1 - C_n \cdot S \cdot Y_t} \qquad (2)$$

The equilibrium frequency of C ($C_e$), namely, the frequency of C when $C_{n+1} = C_n$, is given by:

$$C_e = V/(S \cdot Y_t) \qquad (3)$$

and the increment of the mortality rate at the age t ($\Delta m_t$) will be:

$$\Delta m_t = C_e \cdot S = V/Y_t \qquad (4)$$

Supposing the existence of n t-genes for each age t, all the genes for sake of simplicity with equal values of V (the value of S is irrelevant), at each age:

$$\Delta m_t = n \cdot V/Y_t \qquad (5)$$

namely, $\Delta m_t$ follows the decrement of $Y_t$ (=$\lambda$), increasing slowly until $Y_t$ is very small and only then becoming exponential.

In Fig. 2, curve A (–■–) is the life table of *Rangifer tarandus* (M) (for the simulation, Weibull’s equation, $m_t = m_0 + \alpha \cdot t^\beta$, has been used, with $m_0 = 0.076$, $\alpha = 0.00203$; $\beta = 2.9968$; equation and data are from Ricklefs[54] (see p. 27 and Table A2 in Appendix A); curve B (-▲-) is a hypothetical life table of the same species with only the extrinsic mortality at its lowest value ($m_0$); curve C (··o··) is a hypothetical life table with $m_0$ plus the effects of a great number of t-genes with high mutation rates from their inactive alleles (n = 500; V = 0.00001). Notwithstanding the high values of n, curve C is quite different from curve A and the area between curve A and curve C is completely unjustifiable as effect of t-genes insufficiently eliminated by selection.

The same argument with appropriate modifications can be put forward for recessive t-genes too. In such a case:

$$C_e = \sqrt{(V/(S \cdot Y_t))} \qquad (3')$$

and

$$\Delta m_t = C_e^2 \cdot S = V / Y_t, \qquad (4')$$

as for dominant t-genes.

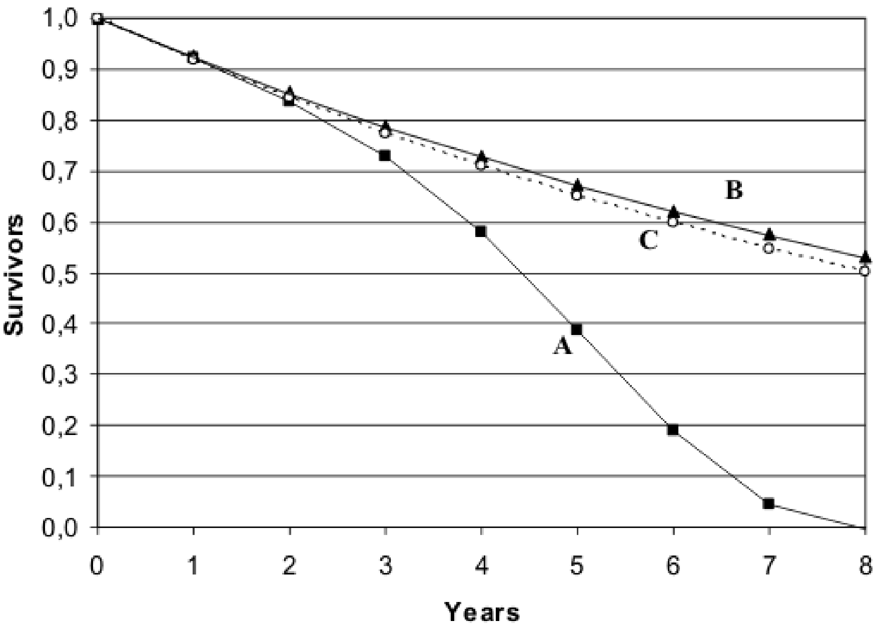

**FIGURE 2**. Effect of many t-genes on a life table (for explanations, see text).

In short, even with extreme conditions, the decline of $Y_t$ in the wild, namely, IMICAW phenomenon, cannot be the consequence of insufficient selection against t-genes and theory A cannot be proposed as an explanation of IMICAW.

Besides these theoretical arguments, data from natural observation severely contradict predictions of theory A.

In fact, for theory A: "The principal determinant in the evolution of longevity is predicted to be the level of extrinsic mortality. If this level is high, life expectancy in the wild is short, the force of selection attenuates fast, deleterious gene effects accumulate at earlier ages, and there is little selection for a high level of somatic maintenance. Consequently, the organism is predicted to be short lived even when studied in a protected environment. Conversely, if the level of extrinsic mortality is low, selection is predicted to postpone deleterious gene effects and to direct greater investment in building and maintaining a durable soma"[40]. But, in a review of IMICAW data for many populations of birds and mammals[54], a significant ($p < 0.0001$) inverse relationship between the proportion of deaths caused by intrinsic mortality ("$P_s$", "senescent deaths", but it would be better to say "IMICAW deaths") and those caused by the extrinsic mortality has been reported.

This result is illustrated in Figs. 3–5, referred to species with different "$m_0$" (small in Fig. 3, intermediate in Fig. 4, and high in Fig. 5; data are from Ricklefs[54]).

For each figure, curve A (-■-) is the life table of the species in the wild, while curve B (-▲-) is a hypothetical life table determined only by $m_0$. The proportion between area Z and the sum of areas Z and W by theory A is predicted to become greater from species to species with higher $m_0$ while data show the opposite (in particular, see Fig. 7 in Ricklefs[54]).

Ricklefs observes (pp. 25–26): "... the analyses reveal that populations with lower extrinsic mortality suffer a higher proportion of senescent deaths. This result is inconsistent with genetic models of senescence based on mutation accumulation and certain types of antagonistic pleiotropy, and it suggests instead that the pattern of senescence balances somatic wear and tear against maintenance and repair mechanisms whose efficacy is under genetic control."; (ibidem, p. 33): "… the maximum fraction of senescent deaths ($P_s$) varies from 79% at $m_0$ = 0.01 yr$^{-1}$ to less than 3% at $m_0$ = 1.0 yr$^{-1}$ … Senescence reduces average life span by only 2% when $m_0$ = 1.0 yr$^{-1}$ but by almost 80% when $m_0$ = 0.01 yr$^{-1}$ ..."; (ibidem, p. 35): "... the observation

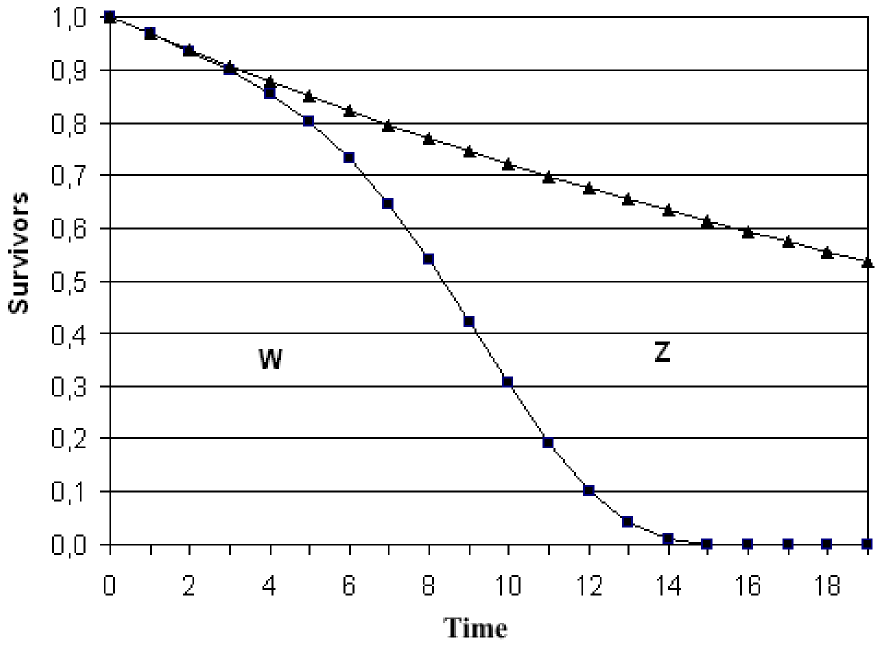

**FIGURE 3.** Life table of *Panthera leo* (F) with (-■-) and without (-▲-) intrinsic mortality. With a low extrinsic mortality ($m_0$, see the slope of curve -▲-) the value of $P_s$ (area Z) is high.

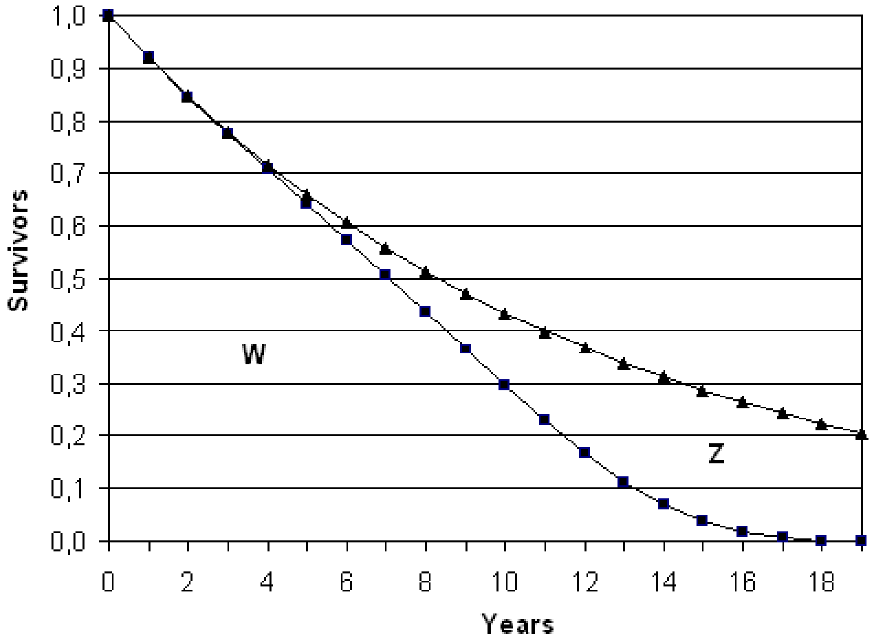

**FIGURE 4.** Life table of *Puffinus tenuirostris* (MF) with (-■-) and without (-▲-) intrinsic mortality. With intermediate extrinsic mortality area Z is of intermediate dimension.

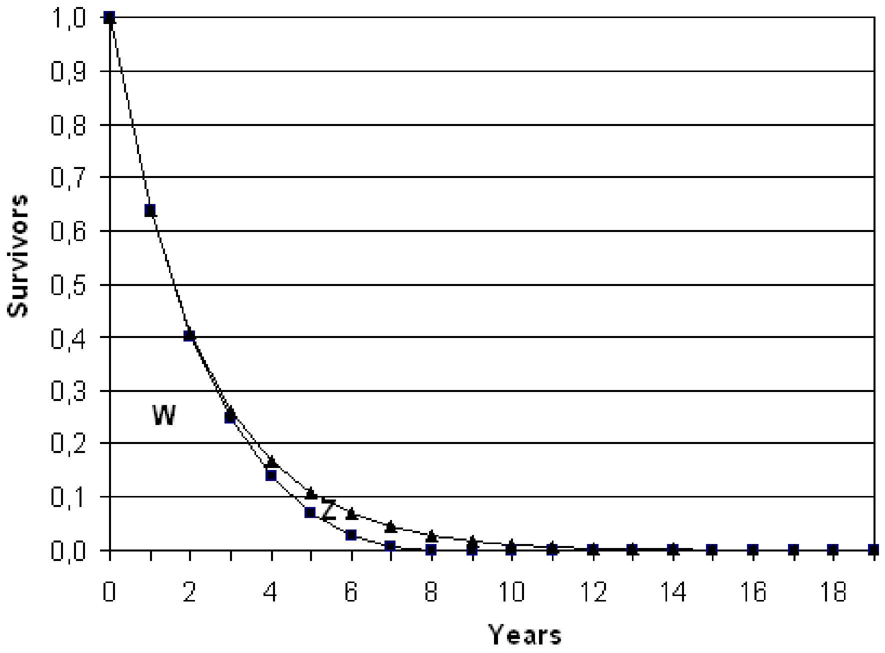

**FIGURE 5.** Life table of *Parus atricapillus* (MF) with (-■-) and without (-▲-) intrinsic mortality. With high extrinsic mortality area Z is minimal.

reported here of increasing senescence-related mortality in populations with progressively older age structure (lower initial mortality rate, $m_0$) weighs against two popular hypotheses (mutation accumulation and antagonistic genetic pleiotropy) for the genetic basis of aging in birds and mammals. The repair hypothesis of senescence ... [*"disposable soma" theory*, *theory C*; n. of A.] could be consistent with observed patterns of aging-related mortality if genetic variation for repair capabilities decreased with increasing age of expression of physiological deterioration. That is, the lesser damage experienced by younger individuals may be more readily prevented or repaired than the more serious damage suffered by older individuals ...".

In seeming contradiction with these statements, data reported in the same paper show a significant positive relationship between $m_0$ and the increment of mortality at increasing chronological ages ("$\Delta m$") and this is interpreted by Ricklefs as a confirmation of the current theories on "aging".

But, the same data show a significant inverse relationship between $m_0$ and the fraction of survivors at the age when the total increment of mortality becomes greater than $m_0$.

Moreover, in the species where $m_0$ is at its lowest values, $\Delta m$ is enough to cause a drastic reduction of the ML by about 80% and a $P_s$ of about 79%, values that are unexplainable with the theory A (see Fig. 3). In other species, with greater values of $m_0$, there is an increase of $\Delta m$ insufficient to increase $P_s$ as predicted by theory A and on the contrary $P_s$ lowers (see Fig. 4). When $m_0$ is at its highest values, $\Delta m$ reaches its greatest values, but ML is minimally reduced (2%) in comparison with a life table having a constant mortality rate equal to $m_0$, and $P_s$ reaches its lowest value (only 3%) (see Fig. 5).

Besides, the extrinsic mortality ($m_e$) is not necessarily a constant and an increment of the mortality due to the effects of the accumulation of injury damages ($m_w$) should be considered, in a sort of wear-and-tear action positively related to the initial extrinsic mortality ($m_0$). Therefore, the overall mortality rate ($m_T$) should be described as the sum of the intrinsic mortality ($m_i$) and of two components of extrinsic mortality:

$$m_T = m_i + m_e = m_i + (m_0 + m_w) \quad (6)$$

However, this $m_w$ factor would be quantitatively important only when $m_0$ is high (ML reduction ≤ 2% and $P_s$ ≤ 3% in Ricklefs' data), while for low values of $m_0$ its influence would be small.

## Theory B

Theoretical arguments expressed against theory A are invalidated with the hypothesis of antagonistically pleiotropic genes with deleterious effects at ages present in the wild. But, theory B like theory A predicts in data from natural observation a direct correlation between $m_e$ and $m_i$ and is similarly disproved by Ricklefs' data, as clearly stated by the same A (see citations reported above).

For some animal models, e.g., *Drosophila melanogaster*, *Caenorhabditis elegans,* and the mouse, the existence of many genes has been proven, the interaction of which alters metabolic pathways, in particular mitochondrial functions, and at the same time increases life span[63]. But, to demonstrate theory B as a valid cause of the IMICAW phenomenon, the same should be proved in wild conditions for IMICAW species and, moreover, we should demonstrate the existence of such genes in a sufficient number to cause the increment of mortality in IMICAW species and their simultaneous absence or insufficient action in non-IMICAW species.

## Theory C

Theoretical arguments against theory A are invalidated as well with the hypothesis of physiological, biochemical, or environmental constraints that limit survival at ages present in the wild.

As for theory A and B, a direct correlation between $m_e$ and $m_i$ is predicted, but disproved, by observational data for IMICAW species. Ricklefs states (see citation above reported) that theory C is consistent with observational data only with a further hypothesis, viz. stronger constraints in the case of lower extrinsic mortality and weaker constraints in the opposite case. But, Ricklefs offers no evidence that this is actually the case.

As regards above-quoted experimental data[63], since the practical distinction between antagonistically pleiotropic genes and physiological, biochemical, or environmental constraints that limit survival is difficult, many of these data could be a valid support of theory C. But, to demonstrate theory C as valid cause of the IMICAW phenomenon, we need similar proofs as those for theory B.

Moreover, the trade-off between reproduction and longevity, a distinctive prediction of theory C, is not proved by available data for human, primates[64] or any other IMICAW species.

## Theory D

This theory was aimed specifically at the evolutionary explanation of IMICAW and the possibility that IMICAW-causing genes could be advantageous was evaluated in terms not of individual, but of "inclusive fitness"[65,66,67].

The theoretical consistency of the arguments was tested with the formulation of a theoretical model and its subsequent analysis. For simplicity, organisms were considered as haploid, asexual, and having discrete generations, specifying that arguments might be formulated for diploid and recombinant organisms too, with unduly useless complications, and that the condition of discrete generations, namely, making a single calculation for each generation as if individuals were living in a synchronous way, is a standard mathematical simplification. In defense of the scientific correctness of the method, Bell's argumentations[68] were mentioned.

First, it was observed that a reduced ML brings about a quicker generation turnover and hence a greater spreading velocity, within the species, of any advantageous mutation, as already expressed by a botanist, Leopold[69], although only in qualitative terms and as an advantage for the species, and long ago by Weismann in intuitive terms, as reported in a review[38]: “[for Weissmann] ... ageing is needed to guarantee or to accelerate the turnover of generations so as to improve a species’ chance of adapting to changes in its environment ...”.

As a matter of fact, the spreading within a species at each succeeding generation (n, n + 1, ...) of a favorable allele C with advantage S in comparison with a neutral allele C’, is described by the simple formula:

$$C_{n+1} = \frac{C_n \cdot (1 + S)}{C_n \cdot (1 + S) + C'_n} = \frac{C_n \cdot (1 + S)}{1 + C_n \cdot S} \qquad (7)$$

Fig. 6 shows the variation of gene C diffusion according to the variation of S values.

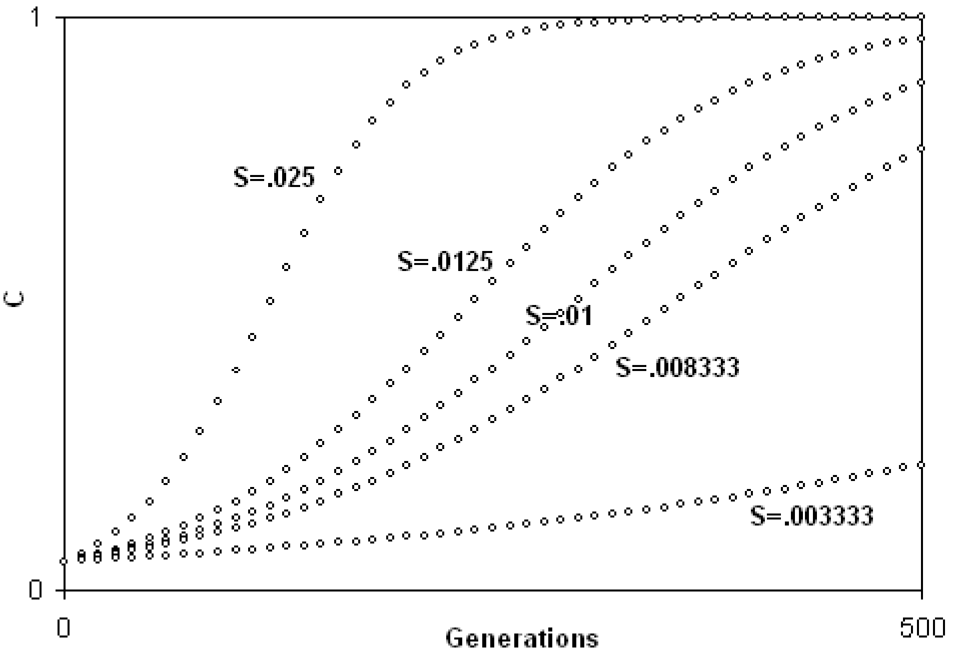

**FIGURE 6.** Spreading of a gene (C) according to the variation of S. The ML, that determines the rate of generation turnover, has a constant value (= 1) for all the five curves. Going from top to bottom, the values of S (arbitrarily chosen) are: $S_1 = 0.025$; $S_2 = 0.0125$; $S_3 = 0.01$; $S_4 = 0.008333$; and $S_5 = 0.003333$. Moreover: $C_o = 0.05$. (This and the following figure are reprinted from [1].)

Now, assuming S constant (= k) and varying ML, namely, varying the rate of generation turnover, with values of $ML_i$ equal to $k/S_i$ (where $S_i$ mean an S value in the curves of the previous figure), we obtain, see Fig. 7, a series of curves morphologically identical to those in Fig. 6.

In quantitative terms, between two populations, other things being equal, an increase of the velocity of generation turnover was shown to be exactly equivalent, as regards the diffusion of any advantageous gene, to a proportional increase of gene advantage[1].

Since the argument expressed in terms of group selection is totally unacceptable[70,71], an IMICAW-causing, i.e., ML-reducing, hypothetical gene C would cause a damage S’ for the individual and the C gene would decay as described by the trivial formula:

$$C_{n+1} = \frac{C_n \cdot (1 - S')}{1 - C_n \cdot S'} \qquad (8)$$

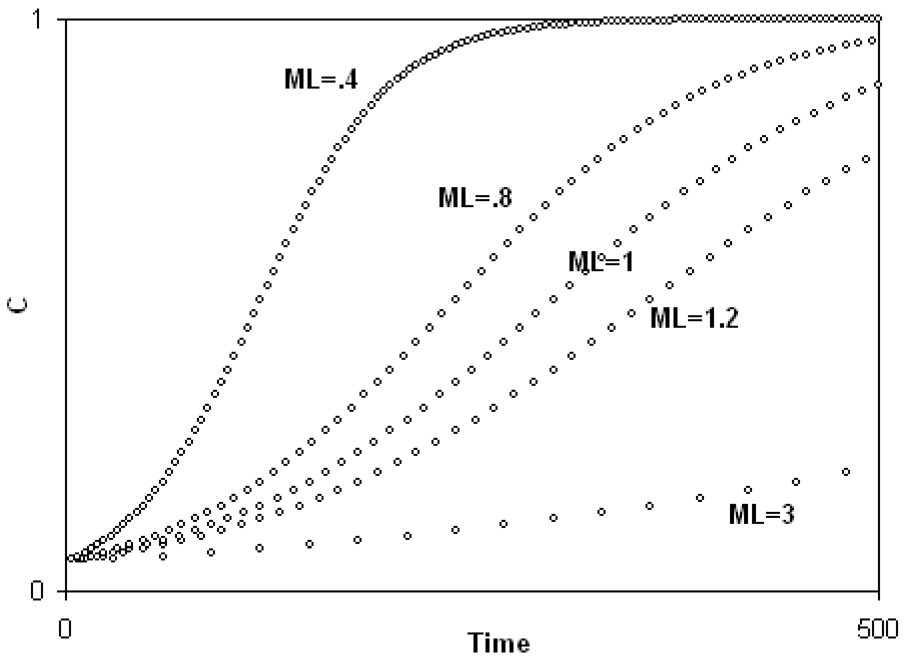

**FIGURE 7.** Spreading of a gene according to the variation of ML. The abscissas indicate the time and not the generations. The advantage S has a constant value (k = 0.01) for all the five curves, while ML has the following different values: $ML_1 = 0.4$; $ML_2 = 0.8$; $ML_3 = 1$; $ML_4 = 1.2$; $ML_5 = 3$. The values of ML are given by the formula: $ML_i = k/S_i$, where $S_i$ indicates an S value in the curves of the previous figure and k is the same value of $S_3$ in the previous figure.

But, it was then observed that individual selection describes only partially the selective pressures determining the frequency variations of a gene since it is indispensable to calculate the inclusive fitness of a gene to predict its exact spreading or decay[65,66,67], with the due specification that kin selection is by no means a sort of group selection theory.

As a matter of fact, the frequency variation ($\Delta_x$) of a gene X present in the individual I will be given by the formula:

$$\Delta_x = \Sigma (S_n \cdot P_n \cdot r_n) \quad (9)$$

where index n goes from 1 (individual I) to z (the last individual for which the action of X has some consequence in terms of fitness), $S_n$ is the advantage or disadvantage for individual n, $P_n$ is the residual reproductive potentiality of individual n, $r_n$ the coefficient of relationship between individual I and individual n, namely, the probability that X is present in individual n (clearly it is equal to 1 in the case of individual I).

Only and exclusively if gene X has no action on the fitness of individuals other than I, does the formula become:

$$\Delta_x = S \cdot P \quad (10)$$

which is the classic concept of Darwinian selection.

On the contrary, if gene X somehow modifies the fitness of individuals other than I, to use the simplified formula is misleading. In particular, to maintain that a mortality-increasing gene, with no other action in the same individual, is always contrasted by natural selection because it is harmful for the individual, is theoretically wrong since this presupposes that it cannot have a positive action for the fitness of other individuals.

It was then observed[1] that the inclusive fitness of an IMICAW-causing, i.e., ML-reducing, hypothetical gene C could be positive if the individuals I predeceased by action of gene C were replaced by an individual (I') with a mean coefficient of relationship (r) between I and I' superior to the mean r of the whole species, namely, in analytical terms when:

$$r \cdot \Sigma(S) \cdot (1/ML_C - 1/ML_{C'}) > S' \quad (11)$$

where $ML_C$ and $ML_{C'}$ are the ML of individuals with the gene C and the allele C', respectively; $\Sigma(S)$ is the summation notation of the advantages of all the favorable genes spreading within the species; S' is the disadvantage of a smaller ML; r is the mean coefficient of relationship between I and I'.

So, the spreading, or decay, within a population of an IMICAW-causing gene C was said to be expressed by the recursion formula:

$$C_{n+1} = \frac{C_n \cdot [1 + r \cdot \Sigma(S) \cdot (1/ML_C - 1/ML_{C'}) - S']}{1 + C_n \cdot [r \cdot \Sigma(S) \cdot (1/ML_C - 1/ML_{C'}) - S']} \quad (12)$$

In ecological terms, in order for the spreading instead of the decay of C to occur, two conditions were shown to be necessarily present:

1. Population numerically constant, as a consequence of a limited living space, so that only when individuals die there is place for new individuals (Pianka[72]: "Population size ... Fairly constant in time, equilibrium; at or near carrying capacity of the environment; saturated communities...").
2. Dying individuals replaced by individuals with a mean r, between them and I', superior to the mean r of the whole population.

The first condition was known to be verified, in general, with K-selected population and to be unrealistic with r-selected species[72], while the second condition was said to prevail for species divided in small demes with a limited intergenic flow, for perennial plants and sessile animals and for territorial species, all conditions usually associated with K selection too. According to theory D, IMICAW phenomenon was therefore expected in conditions of K selection and, on the contrary, not expected in conditions of r selection. These predictions were shown to be generally confirmed by natural observations[67,72]. (For two possible significant objections see Appendix.)

Moreover, since ML is determined both by IMICAW-causing genes, namely, intrinsic mortality ($m_i$), and by extrinsic mortality ($m_e$), generation turnover would be fast with high values of $m_e$ even with no $m_i$ and, hence, in such conditions IMICAW-causing genes would not be advantageous. In the intermediate cases, raising $m_e$ from lower to greater values, IMICAW was expected to be more and more retarded and, therefore, the proportion of ML shortening (namely, $P_s$ in Ricklefs' terminology) determined by $m_i$ would be gradually lowered ("Methuselah effect"[1]). As mentioned above, this apparently paradoxical prediction was the exact opposite of the prediction of theories A–C.

When the theory was published, there was no documentation of the "Methuselah effect" and it was possible to quote only examples of the extreme case in which, with high values of $m_e$, the absence of the IMICAW phenomenon was predicted and verified.

Ricklefs' data show:

1. A splendid documentation of the reality of the "Methuselah effect" with a strong and significant inverse relationship between $m_e$ and the IMICAW-related reduction of ML, i.e., $P_s$ or senescence deaths in Ricklefs terminology.

2. For species with the highest values of $m_0$, Ricklefs reports a $\Delta m$-causing ML reduction $\leq 2\%$ and $P_s \leq 3$. Theory D predicts that with high values of $m_e$, a species should be non-IMICAW, namely, that $m_i$ should be zero (ML-reduction and $P_s = 0$). As discussed above, the $\Delta m$ observed with high values of $m_0$ should not be considered $m_i$ or IMICAW, but an increment of the extrinsic mortality due to the effects of the accumulation of injury damages (see Eq. 6). This factor was not considered in the original formulation of theory D and the present arguments should be considered a clarification of this theory. However, the absence of $m_i$, in the case of high $m_e$ correctly interpreted as sum of $m_0$ and $m_w$, is an important criterion to test the soundness of the theories under review.

In fact, remembering that: "Bidder pointed out that there were several lower vertebrates in which there was no ground for suspecting that the mortality ever increased with increasing age, beyond the inevitable increment from accumulation of evident injuries. .... the disproof of almost all the major existing theories of senescence would follow from the demonstration that is not universally present in the vertebrates." (Comfort[73], pp. 13 and 15), if we restrict mortality increments in Bidder's observation to wild conditions, it is possible to state that an IMICAW-explaining theory should justify the existence of non-IMICAW species too, namely life tables of type II (constant mortality rate for all age groups) and III (very high mortality rate in the beginning of life, then a constant mortality rate)[50,74]. Using this terminology, theories A–C do not satisfy this criterion ("Bidder's criterion"), but, for disposable soma theory, in the particular case when there is no separation between soma and germ line[75], while theory D does so. (Bidder's criterion should not be confused with Bidder's hypothesis, reported and disproved by the same Comfort[73], that "... senescence is a correlate of the evolution of determinate growth and of a final absolute size ... I have suggested that senescence is the result of the continued action of the regulator after growth is stopped...").

## THE FOUR THEORIES TESTED AS EXPLANATION OF THE "STATE OF SENILITY"

Now, in this section, the four theories are evaluated as possible explanation of the "state of senility", namely, with reference to Fig. 1, of the great fitness reduction of the individuals in the area below curve B and on the right side of the line C ("area BC") that is the artificial amplification of the small area below curve A and on the right side of the line C ("area AC").

### Theory A

As expressed with Eq. (3) and (3'), when in the wild, $Y_t$ is very small (see "area AC") with any value of t on the right of line C a t-gene may exist in a nonsmall percentage of $Y_t$, as a consequence of the weakness of the selection against the gene. If for a population of the same species the mortality is drastically lowered (e.g., by captivity or civilization), the fraction $Y_t$ becomes great (see "area BC") and the frequency of the individuals with the t-gene, although unchanged in the fraction $Y_t$, becomes great in the whole population.

With this simple mechanism, it is possible to predict that with an artificial drastic reduction of the mortality, namely, the transformation of area AC in area BC, we should observe a great increment in the whole populations of t-gene–affected individuals, each t-gene, however, regarding only a fraction of the population in area BC and with no difference in the fraction values between the areas AC and BC.

As a matter of fact, for the "state of senility", in current gerontological thought, there is a clear-cut distinction between "damage resulting from intrinsic living processes"[4] or "age changes"[5] and "age-associated diseases"[4,5]. The first category means a process that, whichever are its causes and mechanisms, appears fairly uniform, foreseeable, and quite precisely describable[4]; of course, within the limits of the variety of every biological phenomenon, and regarding all the individuals of a species:

“Aging processes are usually considered to be normal processes in that they occur in all members of the population.”[76].

The second category, limiting the argument to man, includes a fairly heterogeneous array of diseases, the existence of which in evolutionary terms is easily explainable with the aforesaid argument.

On the contrary, phenomena of the first category are not easily explainable as effects of t-genes since “age changes” are present in all the individuals of a species.

### Theory B

It is possible that antagonistically pleiotropic genes with deleterious effect at ages rarely or never present in the wild contribute, similarly to t-genes, to the aforesaid second category of phenomena of the “state of senility”. As for t-genes, phenomena of the first category are not easily explainable as effects of antagonistically pleiotropic genes since “age changes” are present in all the individuals of a species.

### Theory C

Physiological, biochemical, or environmental constraints limiting survival at ages rarely or never present in the wild could explain both first and second category of phenomena of the “state of senility” and even the great variation from species to species of the timing of the “state of senility”. But, an appropriate documentation for IMICAW species is indispensable to transform this hypothesis in a verifiable theory.

### Theory D

For an IMICAW-species, the individuals on the extreme right side of a life table in the wild, with a great reduction of the fitness that is minimally compatible with the wild conditions, alias the individuals in the last period of IMICAW (area AC in Fig. 1), are individuals in the initial phases of the “state of senility” since, by definition, the “state of senility” begins when fitness is low or minimally compatible with wild conditions.

With an artificial lowering of the mortality, the whole life curve is shifted to the right (curve B in Fig. 1), a greater part of the population reaches the age at which individuals are in the initial phases of the “state of senility”, and an ever-increasing fraction of them reaches even older ages at which the manifestations of the “state of senility” are more pronounced and absolutely incompatible with the survival in the wild.

In other terms, disregarding in the “state of senility”, the effects of t-genes insufficiently eliminated by selection, as exposed above for theory A, “age changes” are present in the wild in the few individuals in the area AC and appear to coincide with the effects of the IMICAW-causing mechanisms (not investigated in the aforesaid arguments). Artificially lowering $m_e$, the few individuals become the majority (see area BC in Fig. 1) and some of them reach older or very old age where “age changes” are extreme.

With this interpretation, the greater and extreme manifestations of “age changes”, incompatible with the survival in the wild, are out of control and selectively irrelevant amplifications of physiological although paradoxical effects of the IMICAW-causing mechanisms.

## THE CONCEPT OF IMICAC

For a non-IMICAW species, in conditions of extrinsic mortality lower than in the wild (e.g., in captivity), starting from ages never or very rarely observable in nature when there is no effective selection against harmful genes acting at these ages, the organism is in conditions not influenced by selection and it is predictable that the sum of harmful actions not opposed by selection will cause an increasing mortality with increasing age. This possible increasing mortality with increasing chronological age in captivity ("IMICAC"), for non-IMICAW species and starting from ages never or very rarely observable in nature, was underlined and defined in the same paper in which theory D was proposed[1].

The simple model already used to show that t-genes cannot justify IMICAW phenomenon, gives a good simulation for IMICAC phenomenon. Fig. 8 shows that even a single t-gene for each unity of time should cause a strong increment of mortality rate at ages not existing in the wild. Moreover, considering in the model pleiotropic genes and physiological, biochemical, or environmental constraints with deleterious action at ages not existing in the wild as simulation equivalents to t-genes acting at the same ages, in effect the model includes pleiotropic genes and the constraints described by disposable soma theory too as likely causes of IMICAC.

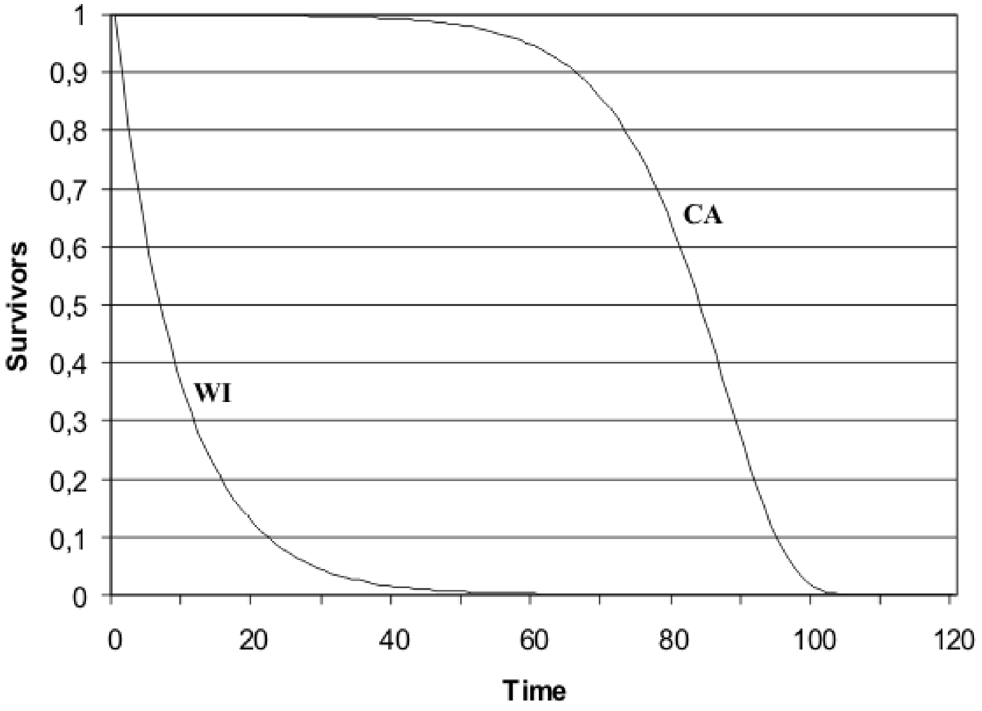

**FIGURE 8.** WI: life table in the wild; CA: life table in captivity. Simulation parameters: extrinsic mortality rate in the wild = 0.1; extrinsic mortality rate in captivity = 0; number of t-genes for each unity of time = 1; mutation rate from an inactive allele to a t-gene = 0.00001.

However, the distinction between IMICAW and IMICAC is by no means a subtle semantic distinction. IMICAW phenomenon is influenced by natural selection and is probably related to "age changes" in IMICAW species, while the hypothesized IMICAC phenomenon is not influenced by natural selection, is present by definition only in non-IMICAW species, and is similar to "age-associated diseases" in IMICAW species.

Another difference to underline is that since IMICAC is conditioned in its timing by life table in the wild, viz. by extrinsic mortality in the wild, IMICAC timing and extrinsic mortality in the wild are predicted as directly related, while IMICAW timing and extrinsic mortality in the wild are predicted as inversely related (Methuselah effect[1]).

As a consequence of all this, studies on IMICAC can clarify IMICAC mechanisms and "age-associated diseases", but would be potentially gravely misleading if intended to explain IMICAW and, likewise, "age changes". In particular, to weigh the studies concerning *Drosophila melanogaster* and *Caenorhabditis elegans*, frequent objects of gerontological research, it should be clarified whether the increment of mortality observed for such species in captivity is a form of delayed IMICAW or, more probably, being r-selected species - IMICAC.

Differences between IMICAW and IMICAC are summarized in table I.

**TABLE 1**
**Differences Between IMICAW and IMICAC**

| IMICAW | IMICAC |
|---|---|
| By definition, IMICAW is observable only in the wild. | By definition, IMICAC is observable only in captivity. |
| IMICAW is influenced by natural selection. | IMICAC cannot be directly influenced by natural selection. |
| IMICAW timing is inversely correlated to extrinsic mortality in the wild. | IMICAC timing is directly correlated to extrinsic mortality in the wild. |
| IMICAW cannot be caused by insufficient selection against t-genes. | IMICAC could be caused by t-genes acting at ages absent in the wild. |
| Pleiotropic genes and the constraints of disposable soma theory, hypothesizing their deleterious effects at ages present in the wild, are not likely causes of IMICAW. | Pleiotropic genes and the constraints of disposable soma theory, hypothesizing their deleterious effects at later ages not existing in the wild, are other likely causes of IMICAC. |

## ANIMALS WITH NEGLIGIBLE SENESCENCE

"Negligible senescence" has been defined the condition of species, such as rockfish, sturgeon, turtles, bivalves, and possibly lobsters[77], showing "no observable increase in age-specific mortality rate or decrease in reproduction rate after sexual maturity; and … no observable age-related decline in physiological capacity or disease resistance"[77].

Restricting the definition of negligible senescence to wild conditions, species with negligible senescence is a synonym of non-IMICAW species.

Since aging for theories A–C is caused by harmful factors cumulating with the passing of time and not sufficiently contrasted by natural selection, the existence of animals reaching very old ages in the wild without any observable decline in their fitness, is a true challenge by no means solved by current theories of aging ("negligible senescence … may be in conflict with mathematical deductions from population genetics theory"[77]). As already underlined, a theory of aging must give a plausible answer to this question in order to be considered as sound (Bidder's criterion).

For theory D, there is a simple prediction: a species that is not in IMICAW-favoring conditions is predicted to be non-IMICAW.

This means that survival in the wild (disregarding possible lesser fitness due to cumulative damages by injuries and, for species with continuous growth, a possible greater fitness due to a greater body mass reducing predation) is described by the simple formula (1): $Y_t = Y_0 \cdot (1 - \lambda)^t$, namely, survival is determined only by the parameter $\lambda$ (extrinsic mortality in the wild). With low values of $\lambda$, it is predicted that, in the wild, some individuals will reach very old ages. In protected conditions, at ages very rarely or never observed in the wild, survival becomes unpredictable and IMICAC phenomenon is possible. As a simple corollary of this phenomenon, for a group of species of the same genus, all in conditions not favoring IMICAW, it is predicted that: (1) all species will be non-IMICAW; (2) in the wild for each species, life span will be in inverse correlation with $\lambda$; (3) in protected conditions for each species, life span will be determined by IMICAC-causing factors and therefore variable from species to species in inverse correlation with λ. Life tables of rockfish genus species are probably a good example of these predictions[78].

## CONCLUSIONS

In this paper, it has been maintained that: (1) both theoretical arguments and observational data are against theories A–C and for theory D as evolutionary explanations of IMICAW; (2) for the "state of senility", theory A and D are plain explanations of "age-associated diseases" and "age changes", respectively.

Indeed, current gerontological thought and the view expressed in this paper represent two different paradigms, in the meaning expressed by Kuhn[79]. The marked differences between the first paradigm[40] and the other paradigm are summarized in Table 2.

**TABLE 2**
**Differences Between the Two Paradigms**

| | First Paradigm | Second Paradigm |
|---|---|---|
| I | The evident alterations of "senescence", in the meaning of "state of senility", are totally or almost incompatible with the survival in the wild. | In the study of evolutionary mechanisms, it is illogical to have as principal object an artificial condition, the "state of senility", totally or almost absent in the wild and, hence, not or little influenced by natural selection. |
| II | So, "senescence", in the meaning of the "state of senility", being nearly absent in the wild, is not object of efficacious selection. | IMICAW, a documented reality, is influenced by natural selection and is, consequently, a proper object for the analysis of evolutionary mechanisms. |
| III | Moreover, "any hypothetical 'accelerating aging gene' would be disadvantageous to the individual. It is therefore difficult to see how genes for accelerated aging could be maintained in stable equilibrium, as individuals in whom the genes were inactivated by mutation would enjoy a selection advantage."[40] | An IMICAW-causing gene reduces the individual fitness, but this does not mean inevitably that the inclusive fitness of the same gene is negative. Only when a gene has no effect on the fitness of other individuals where the gene is present, may the evaluation of the inclusive fitness be disregarded. In the particular case of an IMICAW-causing gene, it has been shown that such a gene may, in certain conditions, have a positive inclusive fitness[1]. |
| IV | Therefore, "senescence" is the outcome of insufficient selection (against harmful genes for theory A, antagonistic pleiotropic genes for theory B, physiological, biochemical, or environmental constraints for theory C). | Therefore, IMICAW is not the result of insufficient selection, but of a balance between positive and negative selecting factors. |
| V | From this, it can be deduced that the less efficacious is the remaining selection, in particular when $m_e$ is greater, the more rapid must be the onset of the "senescence". With $m_e$ at its greatest values, $P_s$ should be at the highest. | From this, it can be deduced that in the case of a weaker favorable selection, e.g., as when $m_e$ is high, an IMICAW-causing gene is less selectively favored and, therefore, $P_s$ is reduced. With $m_e$ at its greatest values, $P_s$ should be zero. These apparently paradoxical predictions, contrary to those of the first paradigm, are confirmed by data from natural observation[54]. |
| VI | There is no explanation for the existence of non-IMICAW species and, indeed, they should not exist for theories A–C (except when there is no separation between soma and germ line[75]). | The existence of non-IMICAW species is predicted in well-defined and common conditions. |

**TABLE 2 (continued)**

| | | |
|---|---|---|
| VII | In short, “senescence”, in the meaning of “state of senility”, is the result of insufficient selection pro a greater longevity and against noxious agents. | “Age changes” in their initial expression coincide with the greatest IMICAW alterations observable in the wild, while in their advanced manifestation are the artificial, by reduction of $m_e$, utmost and unnatural amplification of IMICAW alterations. The “state of senility” is the “age changes” plus the effects of t-genes insufficiently eliminated by natural selection in the wild because of the tardiness of their manifestation (“age-associated diseases”) plus damages by extrinsic factors (category 3 in Masoro’s classification[4]). |
| VIII | So, to contrast “senescence”, identifying the damaging factors (harmful genes, pleiotropic genes, physiological alterations such as oxidant factors, etc.) is an indispensable prerequisite. | Since IMICAW is determined by genes that are in part favored by natural selection, it is necessary to investigate the physiological mechanisms that reduce the fitness in the wild and that cause the “age changes” in conditions of low mortality. The existence of these mechanisms is the main prediction of theory D. On the contrary, for the “age-associated diseases”, plainly interpreted as the outcome of t-genes effects, it is appropriate to act as for other diseases caused by genetic alterations. |
| IX | The life-limiting mechanisms briefly exposed in the Introduction are not predicted by the first paradigm and are hardly compatible with it. | The life-limiting mechanisms briefly exposed in the Introduction are predicted by the second paradigm and are entirely compatible with it. |
| X | The concept of IMICAC is absent and so there is no distinction between IMICAW and IMICAC and no specific care in experimental data evaluation. | The concept of IMICAC is well defined, with a clear distinction between IMICAW and IMICAC, and the necessity of considering this distinction in experimental data evaluation is strongly underlined. |

The main prediction of the “second paradigm” is that IMICAW (and by consequence its artificial amplification in conditions of reduced mortality, i.e., “age changes”) is caused by genetically determined and regulated mechanisms.

Since a theory becomes really vital and valid only when its predictions have sound confirmations in natural observations and experimental data, it has to be wondered whether IMICAW and “age changes” are describable in terms of genetically determined physiopathological mechanisms and whether these are substantiated by known data.

As a matter of fact, it is possible to maintain that the “second paradigm”, as far as its specific physiopathological mechanisms are concerned, has ample confirmations in well-known and accepted data from natural observation and experimentation, as briefly exposed in the Introduction.

After all, when there are two different paradigms, the coexistence in the long run of both is impossible[79] and, therefore, the current paradigm must be reformulated so as to solve satisfactorily the criticisms expressed with the alternative paradigm and the discrepancies between its predictions and data from natural observation. If this is not possible, the current paradigm must be abandoned.

## APPENDIX

For theory D there are two objections needing specific clarifications:

1. The first is that for many IMICAW species (e.g., peregrine birds, cetaceans, many species of herbivorous, etc.) individuals each year emigrate to distant areas, living during migration and in the areas of migration with no territoriality, viz. in conditions which are unfavorable to a C-gene.

But, if individuals of the same species, in mating and reproduction periods, return to well-defined areas, distinct and constant for each group and therefore with characters of territoriality, and if the habitat is saturated in this phase of territoriality, then in this period there are the conditions I and II which favor the spreading of C.

2. The second is that in a deme with a single or few individuals with the gene C, selection will not favor a C-gene since there are not enough copies of C in other individuals that might be benefited by its action. But, on this point the explanation has been already formulated[1]: "as … proposed by Boorman & Levitt (1973) for unselfish genes …, non-selective mechanisms are important up to a critical frequency."

It has to be added that since a random increase of the frequency of a C-gene is plausible in a small population, but improbable in a large one, the division of a species in demes, namely, its territoriality, is a necessary preliminary condition for the spreading of a C-gene in its early phases.

## SUPPLEMENTARY DOCUMENTS

From the Internet address http://www.r-site.org/ageing/simprograms.zip, it is possible to obtain two simulation programs (executable and source files). The first (IMICAW.EXE) simulates the diffusion of a C-gene in certain conditions and its decay in others. The second (IMICAC.EXE) shows the determination of an IMICAC life table by action of t-genes and the impossibility that an IMICAW life table can be caused by t-genes.
